# Morphological and phylogenetic analyses reveal two new *Penicillium* species isolated from the ancient Great Wall loess in Beijing, China

**DOI:** 10.3389/fmicb.2024.1329299

**Published:** 2024-03-15

**Authors:** Ruina Liang, Qiqi Yang, Ying Li, Guohua Yin, Guozhu Zhao

**Affiliations:** ^1^College of Biological Sciences and Technology, Beijing Forestry University, Beijing, China; ^2^National Engineering Research Center of Tree Breeding and Ecological Restoration, Beijing Forestry University, Beijing, China; ^3^College of Biological and Chemical Engineering, Qilu Institute of Technology, Jinan, China

**Keywords:** *Aspergillaceae*, DNA markers, phylogeny, taxonomy, new taxa

## Abstract

**Introduction:**

*Penicillium* species exhibit a broad distribution in nature and play a crucial role in human and ecological environments.

**Methods:**

Two *Penicillium* species isolated from the ancient Great Wall loess in the Mentougou District of Beijing, China, were identified and described as new species, namely, *Penicillium acidogenicum* and *P. floccosum*, based on morphological characteristics and phylogenetic analyses of multiple genes including ITS, *BenA*, *CaM,* and *RPB2* genes.

**Results:**

Phylogenetic analyses showed that both novel species formed a distinctive lineage and that they were most closely related to *P. chrzaszczii* and *P. osmophilum*, respectively.

**Discussion:**

*Penicillium acidogenicum* is characterized by biverticillate conidiophores that produce globose conidia and is distinguished from similar species by its capacity to grow on CYA at 30°C. *Penicillium floccosum* is typically recognized by its restricted growth and floccose colony texture. The description of these two new species provided additional knowledge and new insights into the ecology and distribution of *Penicillium*.

## 1 Introduction

*Penicillium* is widely distributed in various habitats, including soil, plants, air, and indoor settings, and various types of foods (Frisvad and Samson, 2004; Houbraken and Samson, 2011; Visagie et al., 2014a). *Penicillium* fungi, such as *P. rubens* for penicillin production, *P. citrinum* for synthesizing cholesterol-lowering drug mevastatin, *P. camemberti* and *P. roqueforti* for cheese production, and *P. oxalicum* with biocontrol potential, have significant economic value in antibiotic production, pharmaceutical synthesis, biocontrol, food processing, and food safety (Giraud et al., 2010; Houbraken et al., 2011a; Tsang et al., 2018; Steenwyk et al., 2019; Dumas et al., 2020; Yang et al., 2022). In addition, *Penicillium* can have some negative effects in some cases, such as producing a variety of mycotoxins that can cause food contamination and even threaten human health (Frisvad et al., 2004; Perrone and Susca, 2017; Stefanello et al., 2022).

*Penicillium*, established by Link (1809), derives its name from the Latin word penicillus, meaning small brush or paintbrush. The infrageneric classification system of *Penicillium* was mainly based on morphological characteristics in the past 100 years, whereas this phenotype-based sectional classification has been replaced by a system based on a multigene phylogeny in recent decades (Visagie et al., 2014a; Houbraken et al., 2016, 2020). Subgenera, sections, and series are usually transformed from well-supported clades based on DNA sequence analyses. Next-generation sequencing technology has made it possible to obtain a growing number of complete or nearly complete fungal genomes. Phylogenetic analysis based on whole genome sequences to determine the taxonomic position of *Penicillium* and its subordinate members is becoming an important trend in the future (Yang et al., 2016). Currently, 558 species of *Penicillium* were accepted (Wang et al., 2023) and were grouped into two subgenera, namely, *Aspergilloides* and *Penicillium*, 32 sections and 89 series (Houbraken et al., 2020). Species classified in the same section or series share many common features. For example, the series *Canescentia* and *Atroveneta* are closely related in phylogeny, but they can be distinguished by different extrolite profiles and colony textures. Therefore, defining a new species into a section or series could be highly predicted for their functional characteristics (Houbraken et al., 2020).

*Penicillium* section *Citrina* comprises a diverse range of species that exhibit a broad distribution and usually occur in soil habitats. Members of this group are distinguished by their symmetrically biverticillate conidiophores and relatively small, globose to subglobose conidia (Houbraken et al., 2011b; Visagie et al., 2014b; Visagie and Yilmaz, 2023). Furthermore, members of this section have a high potential to produce the mycotoxins citrinin (Houbraken et al., 2011b; Dutra-Silva et al., 2021; Yin et al., 2021). Currently, this section includes 47 species (Houbraken et al., 2020; Andrade et al., 2021; Nguyen et al., 2021; Ashtekar et al., 2022; Tan and Shivas, 2022; Visagie and Yilmaz, 2023). *Penicillium* sect. *Osmophila* was introduced by Houbraken et al. (2016) for species producing bi-, ter-, and quarter-verticillate conidiophores and demonstrating comparable growth rates on CYA when they were incubated at 15 and 25°C. This section currently only contains two species (Houbraken et al., 2020). Members of this section are isolated from soil, and no specific metabolites have been found (Houbraken et al., 2016). Due to the difficulty of delimiting the species within these sections solely based on phenotypic characteristics, a polyphasic approach incorporating morphological, extrolite, genetic data, and multigene phylogenetic analysis has been extensively employed for species identification (Visagie et al., 2014a).

During a survey of *Penicillium* diversity in China, two strains isolated from the ancient Great Wall loess at Qingshui Town, Mentougou District, Beijing, were identified as two new species by multiphase classification. In this study, we provided the morphology of these new species and conducted the phylogenetic analyses using the internal transcribed spacer rDNA area (ITS), partial β-tubulin (*BenA*), calmodulin (*CaM*), and the RNA polymerase II second largest subunit (*RPB2*) genes and compared them with closely related species. The description of these two novel species is expected to enrich our comprehension of *Penicillium* ecology and distribution.

## 2 Materials and methods

### 2.1 Sampling and isolation

Soil samples were collected from loess at the base of the ancient Great Wall (Hongshui Kou section) (39°59′16”N, 115°28′54″E) in Mentougou District, Beijing, China. Cultures were isolated from the soil using the dilution plate method. Initially, 10 g of soil sample was thoroughly mixed with 90 mL of sterile water to prepare a soil suspension. This suspension was then serially diluted to 10^−2^, 10^−3^, and 10^−4^ concentrations. Subsequently, 100 μL of each diluted suspension was plated on potato glucose agar (PDA) with penicillin (50 ppm) and streptomycin (50 ppm) (Lin, 2010). All plates were incubated at 25°C. Type specimens (dry cultures) were deposited in the Fungarium (HMAS), Institute of Microbiology, Chinese Academy of Sciences. Ex-type strains (living cultures) were deposited in the China General Microbiological Culture Collection Centre (CGMCC).

### 2.2 Morphology

Colony characters were observed for strains grown on Czapek yeast autolysate agar (CYA), malt extract agar (MEA), yeast extract sucrose agar (YES), dichloran 18% glycerol agar (DG18), and creatine sucrose agar (CREA). The cultures were incubated at 25°C for 7 days, with extra CYA plates incubated at 30 and 37°C, which are useful for species distinction. Culture media preparation, inoculation technique, and incubation conditions followed the methods described by Visagie et al. (2014a). Color names and codes referred to the Color standards and color nomenclature (Ridgway, 1912). For microscopic observations, slides were made from colonies that have been growing on MEA for 7 days, using phenol glycerin solution as mounting fluid or staining with cotton blue. The isolates were tested for indole metabolite production using the Ehrlich reagent and a filter paper method (Lund, 1995). A violet ring observed within 10 min was considered a positive reaction, while any other color was defined as a negative response (Houbraken et al., 2016).

### 2.3 Observation of scanning electron microscope

Strains were grown for 5–7 days on MEA or PDA, and the conidiophores were mature. Agar blocks (4 × 4 mm) with conidial structures were cut with a blade before transferring to sterile Petri dishes. They were initially fixed with 2.5% glutaraldehyde at room temperature for 2 h, followed by an overnight incubation at 4°C. Subsequently, a gradient dehydration process involved varying ethanol concentrations (30, 50, 70, 95, and 100%), before a transition to tert-butanol (Zhang et al., 2016; Mukherjee et al., 2022). Finally, the samples were freeze-dried, sprayed with gold, and observed using FESEM (Hitachi SU8010, Japan).

### 2.4 DNA extraction, sequencing, and phylogenetic analysis

Strains were grown on PDA for 7 days and DNA was extracted using the E.Z.N.A.® Fungal DNA Mini Kit (Omega Bio-Tek, Inc., United States), involving fungal tissue disruption and lysis, isopropanol precipitation of DNA, precipitation of proteins, and DNA elution. Primers, PCR amplification, and DNA sequencing methods used for the ITS, *BenA*, *CaM*, and *RPB2* genes were based on the description of Visagie et al. (2014a). The newly generated sequences were submitted to GenBank.^1^

Sequence datasets were established using newly generated sequences and reference-type sequences retrieved from GenBank. All datasets were aligned using MEGA 11 implementing the Align by ClustalW option (Tamura et al., 2021). Datasets were analyzed using maximum likelihood (ML) and Bayesian inference (BI). ML analyses were performed within IQtree v. 1.6.12 (Nguyen et al., 2015) and tested by standard non-parametric bootstrap analyses for 1,000 replications (Visagie et al., 2021). The best model for ML was determined using ModelFinder (Kalyaanamoorthy et al., 2017), a fast model-selection method implemented in IQtree. Bayesian inference (BI) analyses were conducted using MrBayes v. 3.2.7 (Ronquist et al., 2012), with a sampling frequency of 100 and the exclusion of the initial 25% of trees as burn-in. The sequences used for phylogenetic analyses in this study are listed in Table 1. Gene sequence alignment datasets were stored in TreeBASE^2^ with the submission number 30834.

**Table 1:** Strains of the genus *Penicillium* used for phylogenetic analyses.

Species	Strain	Substrate and location	GenBank accession numbers			
			ITS	*BenA*	*CaM*	*RPB2*
*P. acidogenicum*	CGMCC3.25421^T^ = CC-1	Soil, Beijing, China	OR512884	OR531524	OR538539	OR538541
*P. allii-sativi*	DTO 149-A8^T^	Bulbs of *Allium sativum* (garlic), Mendoza, Argentina	JX997021	JX996891	JX996232	JX996627
*P. anatolicum*	CBS 479.66^T^	Soil, Turkey	AF033425	JN606849	JN606571	JN606593
*P. argentinense*	CBS 130371^T^	Soil, Valdes Peninsula, prov. Chubet, Argentina	JN831361	JN606815	JN606549	MN969105
*P. atrofulvum*	CBS 109.66^T^	Soil, Katanga near Kipushi, Zaire	JN617663	JN606677	JN606387	JN606620
*P. aurantiacobrunneum*	CBS 126228^T^	Air sample, Cake factory, Give, Denmark	JN617670	JN606702	MN969238	MN969106
*P. cairnsense*	CBS 124325^T^	Soil, Atherton Tableland, Australia	JN617669	JN606693	MN969240	MN969108
*P. carneum*	CBS 112297^T^	Moldy rye bread, Denmark	HQ442338	AY674386	HQ442322	JN406642
*P. christenseniae*	CBS 126236^T^	Soil in the native forest near the base of the aerial tram, Costa Rica	JN617674	JN606680	MN969243	JN606624
*P. chrysogenum*	CBS 306.48^T^	Cheese, Storrs, Connecticut	AF033465	JF909955	JX996273	JN121487
*P. chrzaszczii*	CBS 217.28^T^	Woodland soil, Puszcza Bialowieska Forest, Poland	GU944603	JN606758	MN969244	JN606628
*P. citrinum*	CBS 139.45^T^	Unknown	AF033422	GU944545	MN969245	JF417416
*P. confertum*	CBS 171.87^T^	Cheek pouches of *Dipodomys spectabilis*, Arizona, United States	JX997081	AY674373	JX996963	JX996708
*P. copticola*	CBS 127355^T^	Tortilla, United States	JN617685	JN606817	JN606553	JN606599
*P. cosmopolitanum*	CBS 126995^T^	Heathland soil, Eersel, Cartierheide, The Netherlands	JN617691	JN606733	MN969249	MN969113
*P. decaturense*	CBS 117509^T^	Old resupinate fungus, Ramsey Lake State Park, Decatur, United States	GU944604	GU944604	MN969252	JN606621
*P. desertorum*	DTO 148-I6^T^	Cool desert soil under *Oryzopsis hymenoides*, Wyoming, United States	JX997011	JX996818	JX996937	JX996682
*P. dipodomys*	CBS 110412^T^	Cheek pouches of *Dipodomys spectabilis*, Arizona, United States	MN431359	AY495991	JX996950	JF909932
*P. dokdoense*	JMRC:SF:013606^T^	Soil, Dokdo Island in the East Sea of Korea	MG906868	MH243037	MH243031	n.a.
*P. egyptiacum*	CBS 244.32^T^	Soil, Cairo, Egypt	AF033467	KU896810	JX996969	JN406598
*P. euglaucum*	CBS 323.71^T^	Soil, Argentina	JN617699	JN606856	JN606564	JN606594
*P. expansum*	CBS 325.48^T^	Fruit of *Malus sylvestris*, United States	AY373912	AY674400	DQ911134	JF417427
*P. flavigenum*	CBS 419.89^T^	Flour, Lyngby, Denmark	JX997105	AY495993	JX996281	JN406551
*P. floccosum*	CGMCC3.25422^T^ = CC-2	Soil, Beijing, China	OR512914	OR594640	OR594641	OR538540
*P. gallaicum*	CBS 167.81^T^	Air, Madrid, Spain	JN617690	JN606837	JN606548	JN606609
*P. godlewskii*	CBS 215.28^T^	Soil under pine, Bialowieska, Poland	JN617692	JN606768	MN969258	JN606626
*P. goetzii*	CBS 285.73^T^	Soil, Calgary, Canada	JX997091	KU896815	JX996971	JX996716
*P. gorlenkoanum*	CBS 408.69^T^	Soil, Syria	GU944581	GU944520	MN969259	JN606601
*P. halotolerans*	DTO 148-H9^T^	Salt marsh, Egypt	JX997005	JX996816	JX996935	JX996680
*P. hetheringtonii*	CBS 122392^T^	Soil of beach, Land’s End Garden, Treasure Island, United States	GU944558	GU944538	MN969263	JN606606
*P. kewense*	CBS 344.61^T^	Unknown, Great Britain	AF033466	KU896816	JX996973	JF417428
*P. lanosocoeruleum*	CBS 215.30^T^	Culture contaminant, United States	JX997110	KU896817	JX996967	JX996723
*P. manginii*	CBS 253.31^T^	Unknown	GU944599	JN606651	MN969274	JN606618
*P. mediterraneum*	FMR 15188^T^	Herbivore dung, Balearic Islands, Spain	LT899784	LT898291	LT899768	LT899802
*P. miczynskii*	CBS 220.28^T^	Soil under conifer, Tatra mountains, Poland	GU944600	JN606706	MN969277	JN606623
*P. mononematosum*	CBS 172.87^T^	Heavily molded seed, Amaranthus, Arizona, United States	JX997082	AY495997	JX996964	JX996709
*P. nalgiovense*	CBS 352.48^T^	Ellischauer cheese, Czechoslovakia	AY371617	KU896811	JX996974	JX996719
*P. neomiczynskii*	CBS 126231^T^	Soil, New Zealand	JN617671	JN606705	MN969278	MN969128
*P. nothofagi*	CBS 130383^T^	Soil under Nothofagus, Chile	JN617712	JN606732	JN606507	MN969129
*P. osmophilum*	NRRL 5922^T^	Arable soil, Netherlands	EU427295	MN969391	KU896846	JN121518
*P. pancosmium*	CBS 276.75^T^	Old basidioma of *Armillaria mellea*, on hardwood log; Meech Lake, Gatineau Park, Gatineau County, Quebec, Canada	JN617660	JN606790	MN969284	MN969130
*P. paneum*	CBS 101032^T^	Moldy rye bread, Denmark	HQ442346	AY674387	HQ442331	KU904361
*P. pasqualense*	CBS 126330^T^	Soil, Easter Island, Chile	JN617676	JN606673	MN969286	JN606617
*P. paxilli*	CBS 360.48^T^	Optical instrument, Barro Colorado Island, Panama	GU944577	JN606844	JN606566	JN606610
*P. persicinum*	CBS 111235^T^	Soil, Qinghai, China	JX997072	JF909951	JX996954	JN406644
*P. psychrosexuale*	CBS 128137^T^	Wooden crate in cold-store of apples covered by *Fibulorhizoctonia psychrophile*, the Netherlands	HQ442345	HQ442356	HQ442330	KU904362
*P. quebecense*	CBS 101623^T^	Air in sawmill, Quebec, Canada	JN617661	JN606700	JN606509	JN606622
*P. raphiae*	CBS 126234^T^	Soil under *Raphia*, Las Alturas, Costa Rica	JN617673	JN606657	MN969292	JN606619
*P. restrictum*	CBS 367.48^T^	Soil, Honduras	AF033457	KJ834486	KP016803	JN121506
*P. roqueforti*	CBS 221.30^T^	French Roquefort cheese, United States	KC411724	MN969397	MN969295	JN406588
*P. roseopurpureum*	CBS 226.29^T^	Unknown, Belgium	GU944605	JN606838	JN606556	JN606613
*P. rubens*	CBS 129667^T^	Unknown	JX997057	JF909949	JX996263	JX996658
*P. samsonianum*	CBS 138919^T^	Grassland along the banks of Qumar River, Qinghai, China	KJ668590	KJ668582	KJ668586	KT698899
*P. sanguifluum*	CBS 127032^T^	Soil, Norway	JN617681	JN606819	JN606555	MN969135
*P. shearii*	CBS 290.48^T^	Soil, Tela, Honduras	GU944606	JN606840	EU644068	JN121482
*P. sinaicum*	CBS 279.82^T^	Marine sludge, Egypt	JX997090	KU896818	JX996970	JN406587
*P. sizovae*	CBS 413.69^T^	Soil, Syria	GU944588	GU944535	MN969298	JN606603
*P. steckii*	CBS 260.55^T^	Cotton fabric treated with copper naphthenate, Colorado Island, Panama	GU944597	GU944522	MN969300	JN606602
*P. sucrivorum*	DTO 183-E5^T^	Mite inside infructescence of *Protea repens*, South Africa	JX140872	JX141015	JX141506	MN969140
*P. sumatrense*	CBS 281.36^T^	Soil, Toba Heath, Sumatra, Indonesia	GU944578	JN606639	MN969301	EF198541
*P. tardochrysogenum*	CBS 132200^T^	Soil, McMurdo Dry Valley, Antarctica	JX997028	JX996898	JX996239	JX996634
*P. terrigenum*	CBS 127354^T^	Soil, Hawaii, United States	JN617684	JN606810	JN606583	JN606600
*P. tropicoides*	CBS 122410^T^	Soil of rainforest, Thailand	GU944584	GU944531	MN969303	JN606608
*P. tropicum*	CBS 112584^T^	Soil under *Coffea arabica*, Karnataka	GU944582	GU944532	MN969304	JN606607
*P. ubiquetum*	CBS 126437^T^	Soil, Wilson Botanical Garden, Costa Rica	JN617680	JN606800	MN969306	MN969142
*P. vancouverense*	CBS 126323^T^	Soil under maple tree, Vancouver, British Columbia, Canada	JN617675	JN606663	MN969307	MN969143
*P. vanluykii*	CBS 131539^T^	Lechuguilla Cave, Carlsbad, United States	JX997007	JX996879	JX996220	JX996615
*P. waksmanii*	CBS 230.28^T^	Woodland soil, Puszcza Bialowieska Forest, Poland	GU944602	JN606779	MN969310	JN606627
*P. wellingtonense*	CBS 130375^T^	Soil, Wellington, New Zealand	JN617713	JN606670	MN969311	JN606616
*P. westlingii*	CBS 231.28^T^	Soil under conifer, Denga Goolina, Poznan, Poland	GU944601	JN606718	MN969312	JN606625

## 3 Results

### 3.1 Isolates and identification

Isolations resulted in six fungal isolates obtained from the ancient Great Wall loess, with two suspect new species CC-1(CGMCC 3.25421^T^) and CC-2(CGMCC 3.25422^T^). Through sequencing of ITS, *BenA*, *CaM*, and *RPB2* genes, CC-1(CGMCC 3.25421^T^) generated gene fragments with sizes of 549 bp, 459 bp, 534 bp, and 1,070 bp, and CC-2(CGMCC 3.25422^T^) with sizes of 541 bp, 453 bp, 520 bp, and 1,053 bp, respectively. The blast results of ITS, *BenA*, *CaM*, and *RPB2* genes showed that CC-1 was closely related to *P. chrzaszczii* (Identities: ITS: 99.62%, *BenA*: 95.62%, *CaM*: 96.21%, *RPB2*: 97.27%), while CC-2 was closely related to *P. osmophilum* (Identities: ITS: 98.34%, *BenA*: 96.22%, *CaM*: 95.31%, *RPB2*: 96.97%). Preliminary identification based on blast results of sufficient gene and morphological characteristics, strain CC-1 was designated as a member of *Penicillium* section *Citrina* and strain CC-2 was a member of section *Osmophila*, but neither strain could be identified as any known species, so further phylogenetic analyses were performed.

### 3.2 Phylogenetic analyses

Phylogenetic analyses of the *Penicillium* sections *Citrina*, *Chrysogena*, *Osmophila*, and *Roquefortorum* were conducted using the ITS, *BenA*, *CaM,* and *RPB2* genes, along with a concatenation of the latter three genes, to determine the phylogenetic position of the new species (CGMCC 3.25421^T^ and CGMCC 3.25422^T^). A total of 43 ex-(neo) type strains were involved in the analyses of the individual and combined datasets of section *Citrina*, while 26 ex-(neo) type strains were involved in the analyses of the sections *Chrysogena*, *Osmophila*, and *Roquefortorum*. A summary of the length and substitution models for each dataset is provided in Table 2.

**Table 2:** Length and substitution models for each dataset used in phylogeny.

		Dataset	
		Section *Citrina*	Section *Osmophila* and *Roquefortorum* and *Chrysogena*
ITS dataset	Length (bp)	572	588
	Substitution model (BI)	GTR + I + G	GTR + G
	Substitution model (ML)	TIM2 + F + I + G4	TIM2e + I
*BenA* dataset	Length (bp)	459	437
	Substitution model (BI)	GTR + G	HKY + I + G
	Substitution model (ML)	TIM2e + G4	K2P + G4
*CaM* dataset	Length (bp)	611	505
	Substitution model (BI)	GTR + I + G	K80 + G
	Substitution model (ML)	TIM2e + I + G4	K2P + G4
*RPB2* dataset	Length (bp)	914	960
	Substitution model (BI)	GTR + I + G	SYM + G
	Substitution model (ML)	TNe + I + G4	TNe + G4
Concatenated dataset (*BenA*-*CaM*-*RPB2*)	Length (bp)	1984	1902
	Substitution model (BI)	GTR + I + G	SYM + G
	Substitution model (ML)	TIM2e + I + G4	TNe + G4

#### 3.2.1 Section *Citrina*

**Figure 1:**
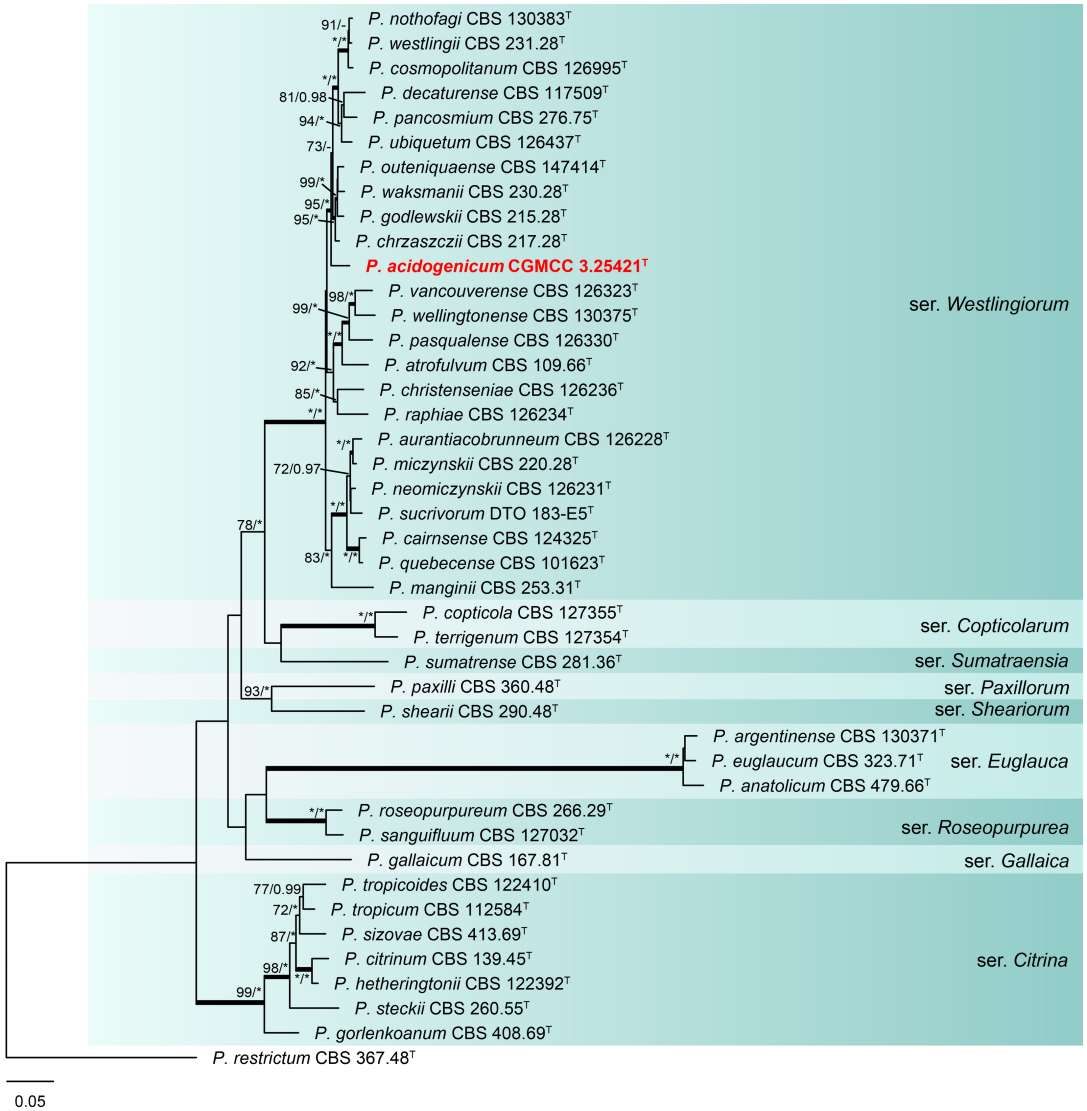
ML phylogenetic tree for concatenated datasets (*BenA*-*CaM*-*RPB2*) of *Penicillium acidogenicum* and *Penicillium* section *Citrina. Penicillium restrictum* was chosen as the outgroup. Bootstrap values (bs) above 70% or posterior probability (pp) above 0.95 are shown at nodes. The branches with more than 95% bs and 1.00 pp values are thickened. Names in red text indicate strains that belong to the new species in this study. *Indicates bs = 100% or pp = 1.00, ^T^ = ex-type strain.

**Figure 2:**
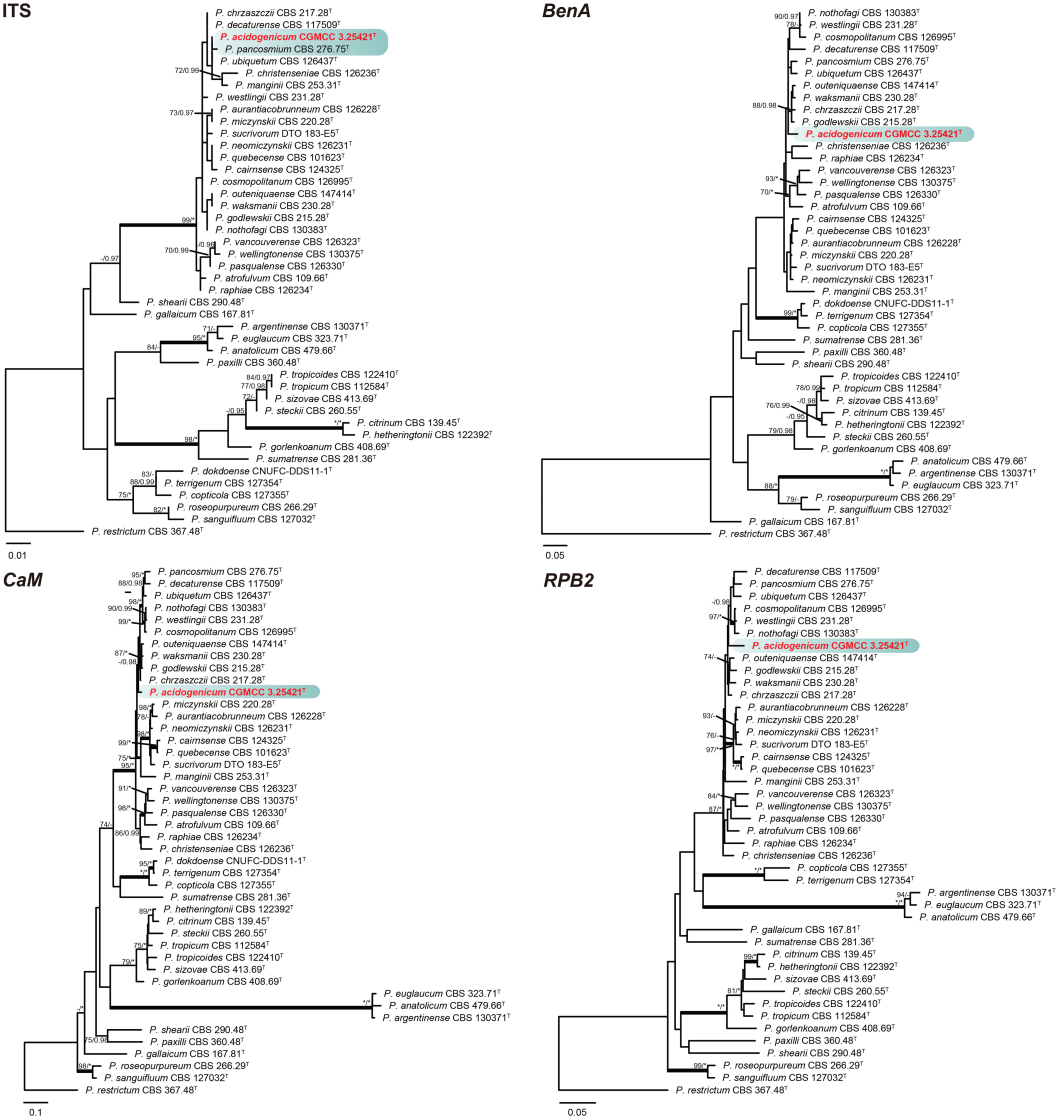
ML phylogenetic tree for individual gene dataset of ITS, *BenA*, *CaM,* and *RPB2* of *Penicillium acidogenicum* and *Penicillium* section *Citrina*. *Penicillium restrictum* was chosen as the outgroup. Bootstrap values (bs) above 70% or posterior probability (pp) above 0.95 are shown at nodes. The branches with more than 95% bs and 1.00 pp values are thickened. Names in red text indicate strains that belong to the new species in this study. *Indicates bs = 100% or pp = 1.00, ^T^ = ex-type strain.

Phylogenetic analyses based on concatenated datasets (*BenA*-*CaM*-*RPB2*) divided section *Citrina* into nine clades (Figure 1), which is consistent with the study by Houbraken et al. (2020). *Penicillium acidogenicum* (CC-1 = CGMCC3.25421^T^) was introduced as a new species, comprising a well-supported distinct clade related to species *P. chrzaszczii* in series *Westlingiorum* (95% bs, 1.00 pp) (Figure 1). The phylogenetic analyses of single-gene revealed that the *CaM* phylogeny better resolved the relationship between these branches in section *Citrina* compared to the ITS, *BenA,* and *RPB2* phylogenies. ITS has poor discriminatory ability in this section. *BenA*, *CaM,* and *RPB2* can easily distinguish the new species, but *BenA* cannot reliably distinguish among *P. decaturense*, *P. pancosmium,* and *P. ubiquetum* (Figure 2).

#### 3.2.2 Section *Chrysogena*, *Osmophila*, and *Roquefortorum*

**Figure 3:**
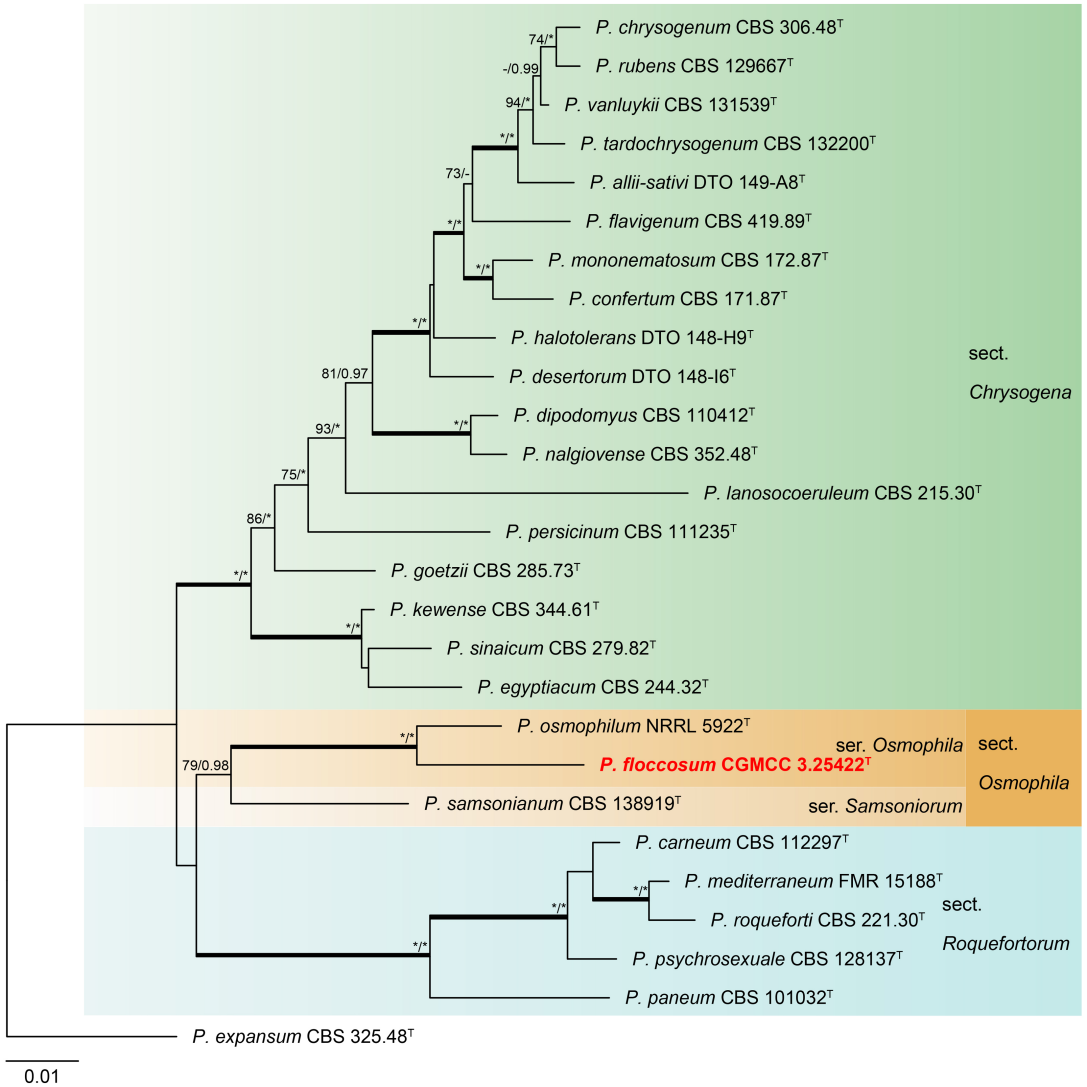
ML phylogenetic tree for concatenated datasets (*BenA*-*CaM*-*RPB2*) of *Penicillium floccosum* and *Penicillium* sections *Chrysogena*, *Osmophila,* and *Roquefortorum*. *Penicillium expansum* was chosen as the outgroup. Bootstrap values (bs) above 70% or posterior probability (pp) above 0.95 are shown at nodes. The branches with more than 95% bs and 1.00 pp values are thickened. Names in red text indicate strains that belong to the new species in this study. *Indicates bs = 100% or pp = 1.00, ^T^ = ex-type strain.

**Figure 4:**
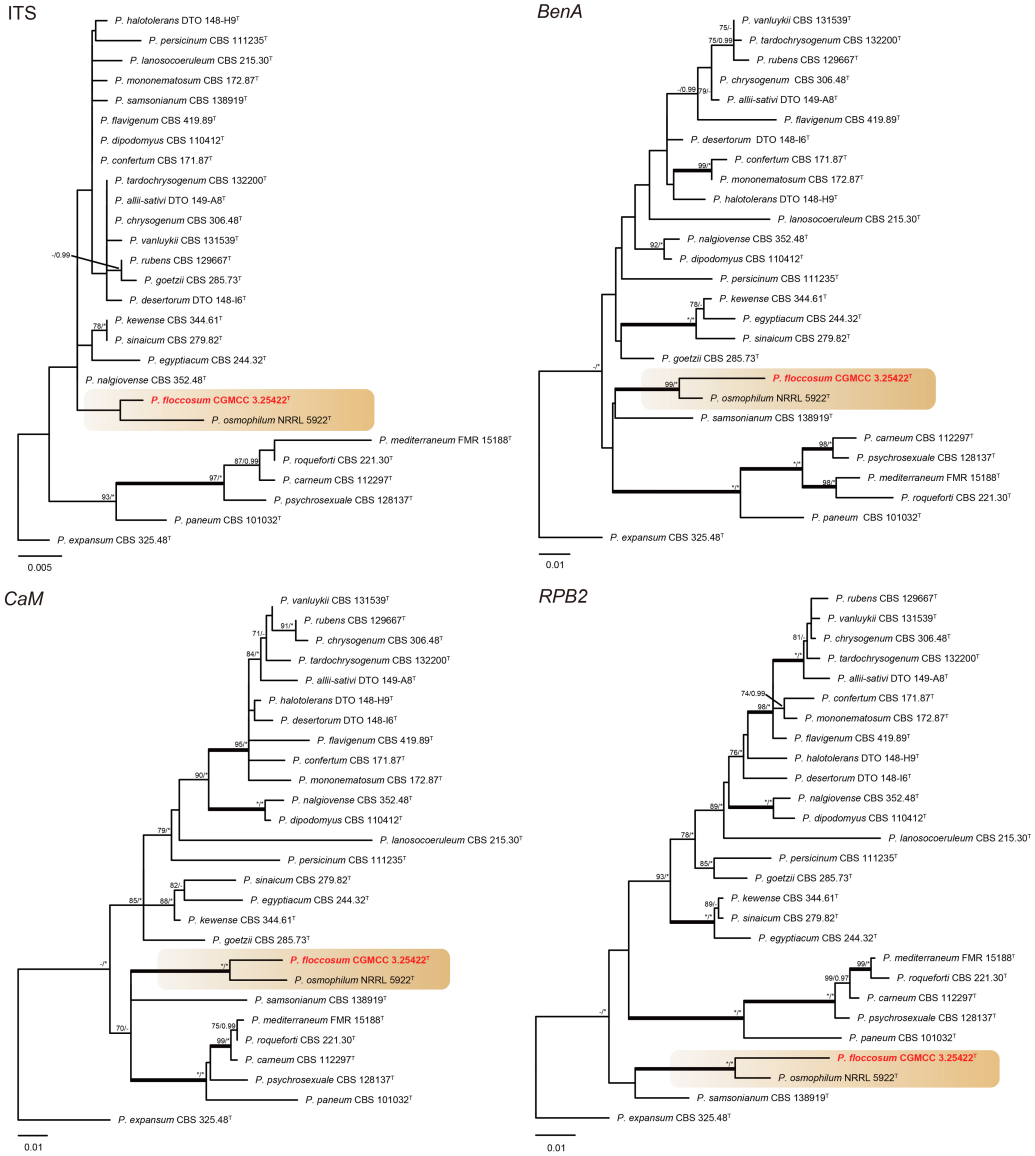
ML phylogenetic tree for individual gene dataset of ITS, *BenA*, *CaM,* and *RPB2* of *Penicillium floccosum* and *Penicillium* sections *Chrysogena*, *Osmophila,* and *Roquefortorum*. *Penicillium expansum* was chosen as the outgroup. Bootstrap values (bs) above 70% or posterior probability (pp) above 0.95 are shown at nodes. The branches with more than 95% bs and 1.00 pp values are thickened. Names in red text indicate strains that belong to the new species in this study. *Indicates bs = 100% or pp = 1.00, ^T^ = ex-type strain.

Phylogenetic analyses revealed a new species in section *Osmophila* and described it as *Penicillium floccosum* (CC-2 = CGMCC3.25422^T^). This species was grouped in a clade as *P. osmophilum* with robust support (100% bs, 1.00 pp) (Figure 3). In the single-gene phylogenies, *P. floccosum* and *P. osmophilum* formed a clade with a high degree of support, except for ITS (Figure 4). Compared with ITS, *BenA*, *CaM,* and *RPB2* can easily distinguish all species of these sections.

### 3.3 Taxonomy

#### 3.3.1 *Penicillium acidogenicum* R. N. Liang and G. Z. Zhao, sp. nov.

Figure 5

**Figure 5:**
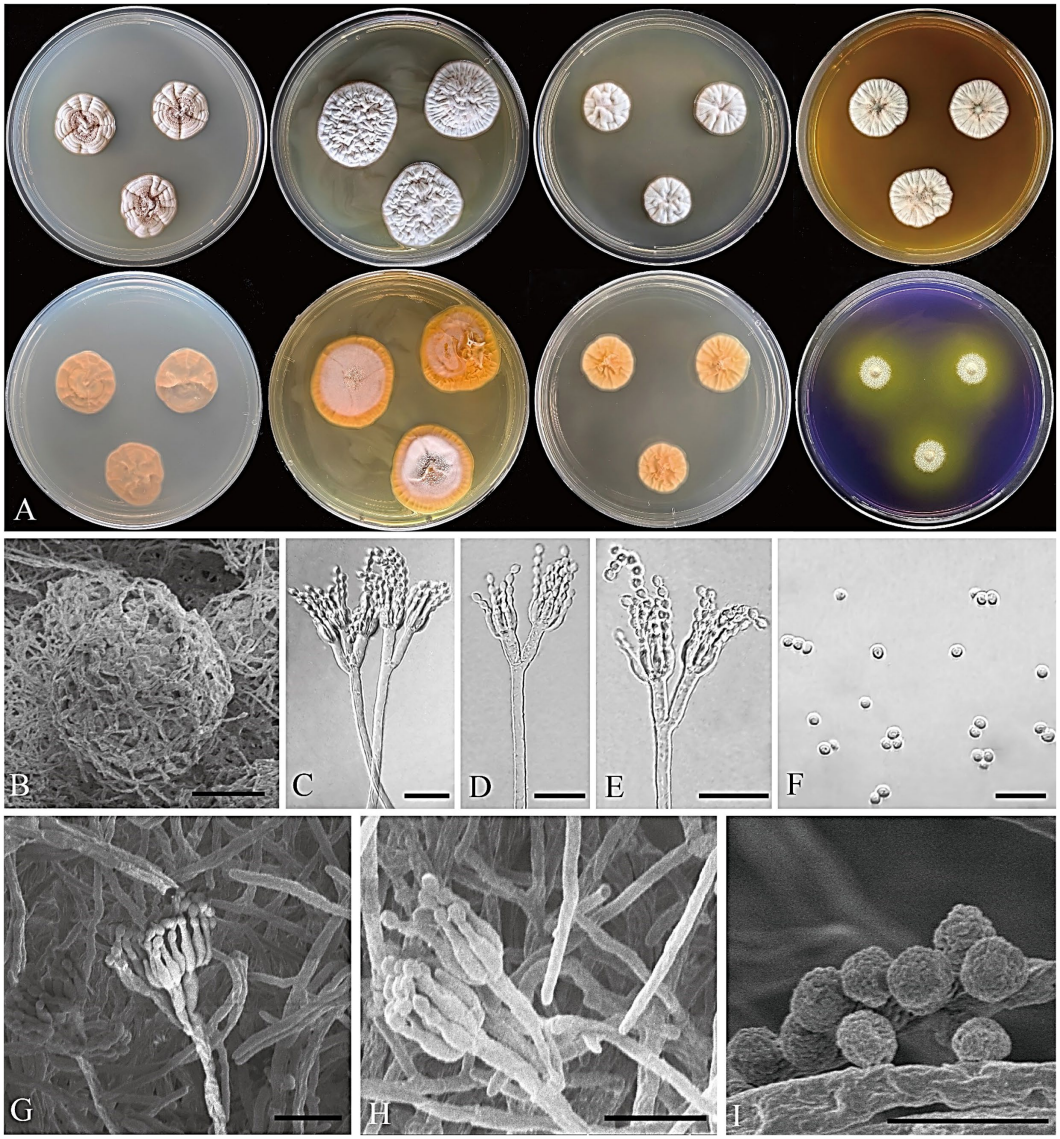
*Penicillium acidogenicum* CGMCC 3.25421. **(A)** Colonies on medium for 7 days (left to right, first row: CYA, YES, DG18, and MEA obverse; second row: CYA, YES, DG18 reverse, and CREA obverse); **(B)** SEM micrograph of cleistothecia; **(C–E)** Conidiophores; **(F)** Conidia; **(G,H)** SEM micrograph of conidiophores; **(I)** SEM micrograph of conidia. Scale bars: B = 50 μm, C–H = 10 μm, I = 5 μm.

MycoBank number: 850530.

Infrageneric classification: subgenus *Aspergilloides*, section *Citrina*, series *Westlingiorum*.

Etymology: “*acidogenicum*” refers to the acid-producing characteristics of colonies grown on CREA.

Type: CHINA. Beijing, Mentougou District, Qingshui Town, from the ancient Great Wall loess, 27 August 2022, collected by G. Z. Zhao, CC-1 (holotype HMAS 352643, dried culture; culture ex-type CGMCC 3.25421).

Colony diameter after 7 days (mm): CYA 18–21; MEA 20–23; YES 24–29; DG18 16–19; CREA: 10–13; CYA 30°C 14–16; CYA 37°C no growth.

Colony characteristics (7 days): CYA at 25°C: Colonies moderately deep, sulcate, slightly elevated at the center; margins entire; mycelium white; texture floccose and funicolose; sporulation moderate, conidia light grayish vinaceous (R. Pl. VIII); exudate clear; reverse light orange-yellow (R. Pl. LXXI); soluble pigment absent. CYA at 30°C: Colonies moderately deep, sulcate, slightly elevated at the center; margins entire; mycelium white; texture floccose and funicolose; sporulation moderate, conidia light grayish vinaceous (R. Pl. XXXII) to gray; exudate clear; reverse orange-pink (R. Pl. LII); soluble pigment absent. MEA at 25°C: Colonies moderately deep, radially sulcate; margins entire; mycelium white; texture floccose; sporulation sparse, conidia livid pink (R. Pl. IV); exudate clear; reverse cadmium orange (R. Pl. XLVIII); soluble pigment absent. YES at 25°C: Colonies moderately deep, sulcate, elevated at the center; margins entire; mycelium white; texture floccose; sporulation sparse to absent; exudate absent; reverse orange (R. Pl. L); soluble pigment absent. DG18 at 25°C: Colonies moderately deep, sulcate, raised at the center; margins entire; mycelium white; texture floccose; sporulation sparse to absent; exudate absent; reverse pale orange-yellow (R. Pl. LXX); soluble pigment absent. CREA at 25°C: weak growth, moderate acid production. Ehrlich reaction negative.

Micromorphology: Cleistothecia produced on CYA and MEA, 80–130 μm diam; conidiophores biverticillate; stipes smooth-walled, 80–235 × 2.5–3.5 μm; metulae divergent, 2–4 per stipe, 8–13 × 1.5–3 μm; phialides ampulliform, 4–8 per metula, 5.5–7.5 × 2–2.5 μm; conidia globose, finely rough, 2–3 μm diam.

Notes: Phylogenetic analyses clustered *Penicillium acidogenicum* within a sister clade alongside 10 species, including *P. chrzaszczii*, *P. godlewskii*, *P. waksmanii*, *P. outeniquaense*, *P. ubiquetum*, *P. pancosmium*, *P. decaturense*, *P. cosmopolitanum*, *P. westlingii,* and *P. nothofagi*. The new species *P. acidogenicum* is phylogenetically most closely related to *P. chrzaszczii*, *P. outeniquaense*, *P. waksmanii,* and *P. godlewskii*. However, the former (colony 14–16 mm diam) can grow on CYA at 30°C, and the latter four cannot grow at 30°C. *Penicillium acidogenicum* can produce acid on CREA which is easily distinguished from the non-acid-producing character of closely related species (Table 3). The growth of *P. acidogenicum* is more restricted on DG18 (colony 16–19 mm) than *P. chrzaszczii* (20–27 mm), *P. outeniquaense* (20–21 mm), and *P. waksmanii* (16–27 mm) (Houbraken et al., 2011b; Visagie and Yilmaz, 2023).

**Table 3:** Morphological features for new species and their closely related species.

Species	Growth rates (in mm)				Conidiophores branching	Cleistothecia/sclerotia	Conidia		References
	CYA	CYA 30°C	CYA 37°C	MEA			Shape	Roughening	
*P. chrzaszczii*	25–33	No growth	No growth	21–28	Symmetrically biverticillate	Absent	Globose to subglobose	Finely rough	Houbraken et al. (2011b)
*P. outeniquaense*	25–27	Germination	No growth	18–19	Biverticillate/mono- or terverticillate rare	Absent	Globose	Finely rough	Visagie and Yilmaz (2023)
*P. godlewskii*	15–25	No growth	No growth	12–20	Symmetrically biverticillate	Absent	Globose to subglobose	Finely rough	Houbraken et al. (2011b)
*P. waksmanii*	(20–) 25–32	No growth	No growth	18–24(−30)	Symmetrically biverticillate	Absent	Globose to subglobose	Finely rough	Houbraken et al. (2011b)
*P. acidogenicum*	18–21	14–16	No growth	20–23	Biverticillate	Cleistothecia	Globose	Finely rough	This study
*P. osmophilum*	14–26	n.a.	n.a.	35 (2 weeks)	Biverticillate-divaricate/monoverticillate rare	Cleistothecia	Broadly ellipsoidal or subglobose	Smooth	Stolk and Veenbaas-Rijks (1974), Pitt (1979), and Houbraken et al. (2016)
*P. floccosum*	24–27	10–13	No growth	24–27	Terverticillate	Absent	Globose	Smooth	This study

#### 3.3.2 *Penicillium floccosum* R. N. Liang and G. Z. Zhao, sp. nov.

Figure 6

**Figure 6:**
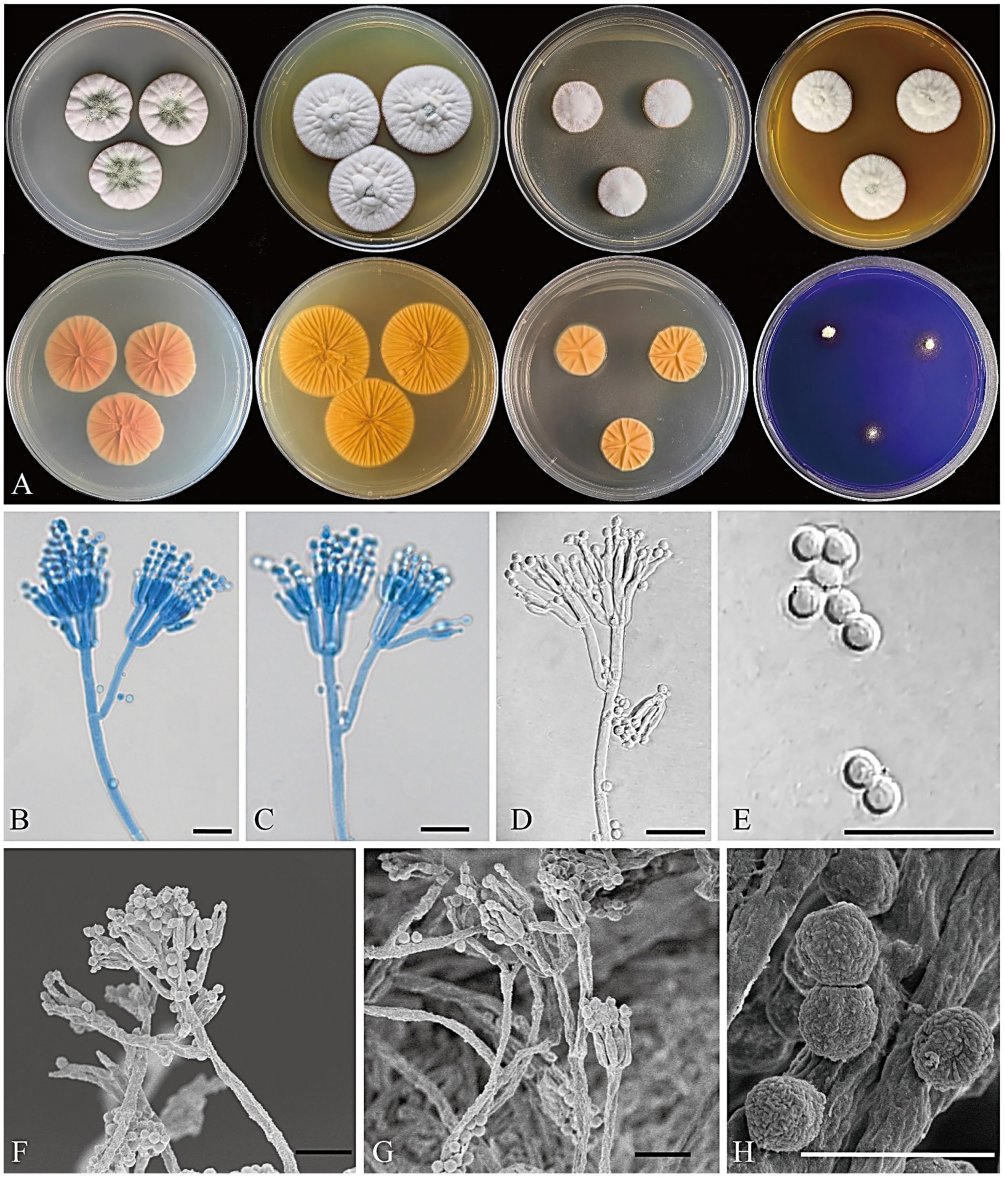
*Penicillium floccosum* CGMCC 3.25422. **(A)** Colonies on medium for 7 days (left to right, first row: CYA, YES, DG18, and MEA obverse; second row: CYA, YES, DG18 reverse, and CREA obverse); **(B–D)** Conidiophores; **(E)** Conidia; **(F,G)** SEM micrograph of conidiophores; **(H)** SEM micrograph of conidia. Scale bars: B–G = 10 μm, H = 5 μm.

MycoBank number: 850534.

Infrageneric classification: subgenus *Penicillium*, section *Osmophila*, series *Osmophila*.

Etymology: “*floccosum*” refers to its floccose colony texture.

Type: CHINA. Beijing, Mentougou District, Qingshui Town, from the ancient Great Wall loess, 27 August 2022, collected by G. Z. Zhao, CC-2 (holotype HMAS 352644, dried culture; culture ex-type CGMCC 3.25422).

Colony diameter after 7 days (mm): CYA 24–27; MEA 24–27; YES 31–34; DG18 18–19; CREA: 5–8; CYA 30°C 10–13; CYA 37°C no growth.

Colony characteristics (7 days): CYA at 25°C: Colonies moderately deep, radially sulcate, elevated at the margin; margins entire; mycelium white; texture floccose; sporulation moderate, conidia dark greenish glaucous (R. Pl. CXXXV); exudate clear; reverse peach red (R. Pl. XXXVII); soluble pigment absent. CYA at 30°C: Colonies moderately deep, radially sulcate, elevated at the center; margins entire; mycelium white; texture floccose; sporulation absent; exudate absent; reverse peach red (R. Pl. XXXVII); soluble pigment absent. MEA at 25°C: Colonies moderately deep, radially sulcate, elevated at the center; margins entire; mycelium white to pale yellow; texture floccose; sporulation sparse, conidia tea green (R. Pl. CXXII); exudate absent; reverse orange to cadmium orange (R. Pl. L); soluble pigment absent. YES at 25°C: Colonies moderately deep, radially sulcate, elevated at center; margins entire; mycelium white; texture floccose; sporulation sparse, conidia cadet gray (R. Pl. CLXXXV) to calamine blue (R. Pl. CLXXI); exudate absent; reverse cadmium yellow (R. Pl. LXVIII) to cadmium orange (R. Pl. L); soluble pigment absent. DG18 at 25°C: Colonies moderately deep, radially sulcate, raised at the center; margins entire; mycelium white; texture floccose; sporulation absent; exudate absent; reverse light orange–yellow (R. Pl. LXXI); soluble pigment absent. CREA at 25°C: weak growth, no acid production. Ehrlich reaction negative.

Micromorphology: Conidiophores terverticillate; stipes smooth to nearly smooth-walled, 70–300 × 2–4 μm; rami 11–21 × 1.5–3 μm; metulae divergent, 2–4 per branch/ramus, 8–12 × 1.5–2.5 μm; phialides ampulliform to cylindrical, 2–6 per metula, 6–7.5 × 1.5–2.5 μm; conidia globose, smooth, 2–3.5 μm diam.

Notes: *Penicillium floccosum* is classified in the section *Osmophila* and phylogenetically closely related to *P. osmophilum*. However, *P. osmophilum* produces ascomata, a feature lacking in the new species. *Penicillium floccosum* produces globose conidia and is distinguished from *P. osmophilum* which produces pear-shaped to ellipsoidal, occasionally subglobose conidia (Table 3; Stolk and Veenbaas-Rijks, 1974).

## 4 Discussion

The delimitation of *Penicillium* species currently relies on a polyphasic approach, typically including morphological characteristics, extrolites data, and multigene phylogenetic analyses. DNA sequence markers used for identification and phylogeny include ITS, *BenA*, *CaM,* and *RPB2* genes (Houbraken et al., 2020). The ITS region is widely recognized as a universal barcode for fungi (Schoch et al., 2012). Nevertheless, in *Penicillium*, ITS is inadequate to distinguish between all closely related species, and secondary markers including *BenA*, *CaM,* and *RPB2* genes are often needed to identify isolates to species accurately (Visagie et al., 2014a). *BenA* offers accurate identification of *Penicillium* species as do *CaM* and *RPB2* (Visagie et al., 2016). Furthermore, *RPB2* contains almost no introns, making it robust and easy to align for phylogenies (Vetrovsky et al., 2016). However, the *RPB2* gene is usually difficult to amplify, probably because the *RPB2* gene sequence varies significantly among different fungal species, and thus, the universal primers contain some degenerate bases that reduce the specificity of PCR amplifications (Liu et al., 1999; Diao et al., 2019). To enhance the all-inclusiveness of the *RPB2* database, it is possible to design primers with higher specificity targeting *Penicillium* and reduce the generation of non-specific amplification products. On the other hand, cloning of the amplification products and sequencing of the recombinant plasmids can be performed to obtain the target gene sequence (Zhou and Gomez-Sanchez, 2000).

In this study, we introduced two new species *Penicillium acidogenicum* and *P. floccosum*, belonging to sections *Citrina* and *Osmophila*, respectively, based on a polyphasic approach. The morphological characteristics of the new species and their closely related species are summarized in Table 3. Section *Citrina* members are frequently found in soil and have also been recovered from indoor environments and food products (Samson et al., 2010; Houbraken et al., 2011b). This shows that members of the group have a relatively wide range of habitats. Accurate species identification is crucial for section *Citrina* strains. Some members of this group produce mycotoxins citrinin, which is widely recognized as a harmful contaminant in food and feed (Houbraken et al., 2010; Gao et al., 2013). For example, Schmidt-Heydt et al. (2019) performed whole genome sequencing on *P. citrinum* DSM 1997 and revealed the biosynthesis gene cluster for citrinin. Section *Osmophila* currently includes three species, *P. osmophilum*, *P. samsonianum*, and the new species *P. floccosum* identified in this study. Species in this group have restricted colony growth with smooth-walled conidiophores and conidia (Stolk and Veenbaas-Rijks, 1974; Houbraken et al., 2016) and are mostly isolated from soil, while *P. osmophilum* is also isolated from the roots of the plant *Colobanthus quitensis* (Hereme et al., 2020).

Soil fungi, distinguished by their abundance and diversity, play a fundamental role in the ecosystem. A few Ascomycota taxa including *Penicillium* species dominate the soil fungal communities (Egidi et al., 2019). The discovery of two new species from the ancient Great Wall loess in Beijing predicts that there may still be a large number of undescribed species in special soil habitats. Therefore, using a polyphasic approach to study *Penicillium* from the soil will enrich the species diversity of the genus and provide more ideas and insights for us to understand the function of fungi in the ecosystem.

## Data availability statement

The datasets presented in this study can be found in online repositories. The names of the repository/repositories and accession number(s) can be found in the article/supplementary material.

## Author contributions

RL: Data curation, Formal analysis, Investigation, Visualization, Writing – original draft. QY: Investigation, Visualization, Writing – original draft. YL: Investigation, Visualization, Writing – original draft. GZ: Conceptualization, Funding acquisition, Methodology, Resources, Supervision, Validation, Writing – review & editing. GY: Conceptualization, Methodology, Resources, Supervision, Validation, Writing – review & editing.

## Footnotes

^1^ www.ncbi.nlm.nih.gov

^2^ www.treebase.org
